# Definitive radiotherapy for early stage glottic cancer by 6 MV photons

**DOI:** 10.1186/1758-3284-4-23

**Published:** 2012-05-18

**Authors:** Chi-Chung Tong, Kwok-Hung Au, Roger Kai-Cheong Ngan, Foon-Yiu Cheung, Sin-Ming Chow, Yiu-Tung Fu, Joseph Siu-Kei Au, Stephen Chun-Key Law

**Affiliations:** 1 Department of Clinical Oncology, Queen Elizabeth Hospital, 30 Gascoigne Road, Kowloon, Hong Kong

**Keywords:** T1/T2N0 glottic cancer, Radiotherapy, 6MV photons, Biologically effective dose

## Abstract

**Purpose:**

To evaluate the clinical outcome of early glottic cancer (GC) treated by primary radiotherapy (RT) with 6 MV photons.

**Methods and materials:**

We retrospectively reviewed the medical records of 695 consecutive patients with T1N0 and T2N0 GC treated between 1983 and 2005 by RT in our institution. Clinical outcome in terms of local control (LC), overall survival (OS) and cause- specific survival (CSS) rate were evaluated.

**Results:**

The median follow-up time was 10.5 years. The 10-year actuarial LC rates were as follows: T1A, 91%; T1B, 87%; T2, 77%. The 10-year OS were as follows: T1, 74.2%; T2, 70.7%. The 10-year CSS were as follows: T1, 97.7%; T2, 97.1%.

Poorly differentiated histology and tumor biologically effective dose < 65 Gy_15_ were adverse factors in both LC of T1 and T2 disease. Involvement of anterior commissure was an adverse factor in both LC and CSS of T1 disease. Subglottic extension was associated with poor LC in T2 disease whereas hemoglobin <13.0 was associated with poor LC and CSS of T2 disease.

**Conclusion:**

Primary RT remains an option among the various standard treatments for early GC. Clinical treatment outcome by 6MV photons is similar and comparable to historic data of Cobalt-60 and 2 MV photons.

## Introduction

Laryngeal cancer is the third most common head and neck (H&N) cancer in Hong Kong. According to Hong Kong Cancer Registry, the age-standardized incidence rate was 2.3 per 100,000 in 2007[1].

In western countries, definitive radiotherapy (RT) and conservative surgery (endoscopic laser surgery/open organ preserving surgery) are accepted standard treatment modalities for early stage laryngeal cancers. The axiom of treatment aims for cure with laryngeal preservation and maintain optimal quality of life like voice quality and swallowing. The choice of treatment depends on availability of facilities and medical expertise, as well as patient’s preference and the cost of treatment [2,3].

A survey conducted to eleven Asian regions/countries about management strategy of early laryngeal cancer revealed that around half of the countries/regions, especially those follow the British stream, like Hong Kong and Singapore, primary radiotherapy remained a mainstay treatment modality [4]. In Hong Kong, around 95% of early glottic cancer (GC) patients were treated by primary radiotherapy (RT) alone.

For definitive RT, there is extensive published data regarding management of early GC with Cobalt-60 or 2-4 MV photons. Factors studied for prognostic importance for local failure included pretreatment hemoglobulin [5], sex [5,6], T category [5,7], histology differentiation [7], anterior commissure involvement [6], subglottic extension [5], tumor bulk [5,6,8], fraction dose size [7] and overall treatment time [7].

The reported treatment outcome of early GC by primary RT with 6MV photons is limited. We present our institution’s experience in this report.

## Methods and materials

### Patient characteristics

In Hong Kong, most citizens are not covered by medical insurance and their medical care is provided mainly by the public care system funded by the Government. The Queen Elizabeth Hospital is one of the major public tertiary referral centers.

In May 2010, we conducted a retrospective review of laryngeal cancer patients referred to our center over a 23 year period for radical treatment between January 1983 to December 2005. A total of 1256 consecutive patients were identified. According to the Hong Kong Cancer Registry, about a quarter of laryngeal cancer cases diagnosed in Hong Kong over that period had been treated in our institution.

Out of the 1256 patients, there were 695 previously untreated patients with T1N0 and T2N0 GC. Six hundred and sixty- two were male and 33 female patients, with a male: female ratio of 20:1. Six hundred and eighty two (98%) were Chinese and 13 (2%) came from other ethic backgrounds. Age ranged from 35 to 94 (median: 65). As the treatment of choice at our institution for stage I and II GC has always been primary RT alone, this represented a relatively unselected cohort of patients.

### Staging

All patients had full physical examination, routine blood counts, renal and liver function tests, chest x ray, endoscopic examination and biopsy for histology diagnosis. Computed tomography (CT) scan of larynx and neck was performed in 661 (95%) patients. All patients were restaged according to TNM 2002 classification [9]. Table 1 summarizes the patient and tumor parameters.

**Table 1:** Patient and tumor parameters

**Parameter**	**T1N0**	**T2N0**
	**Patient no. (%)**	**Patient no. (%)**
All stages	433	262
T1a	324 (74.8%)	-
T1b	109 (25.1%)	-
Gender		
Male	413 (95.3%)	249 (93.8%)
Female	20 (4.6%)	13 (4.9%)
Grade		
Well differentiated	154 (35.5%)	64 (24.4%)
Mod differentiated	273 (63.0%)	179 (68.3%)
Poorly differentiated	6 (1.3%)	19 (7.2%)
Anterior commissure involved		
Yes	197 (45.4%)	186 (71%)
No	236 (54.1%)	76 (29%)
Impaired mobility		
Yes	Not apply	51 (19.4%)
No		211 (80.5%)
Sub-glottic extension		
Yes	Not apply	80 (31%)
No		182 (69%)
Hb level		
< 13 g/dL	45 (10.4%)	28 (10.6%)
≥ 13 g/dL	388 (89.6%)	234 (91.4%)

*Abbreviations:* no. = number; Hb = Hemoglobin.

### Radiotherapy treatment

All patients were treated exclusively with 6-MV photons. They were treated in a supine position, immobilized with a customized neck cast. All patients received a continuous course of RT with once-daily fractionation, 5 fractions per week. All fields are equally weighted and treated in each fraction. Appropriate wedge filters were used to improve the dose homogeneity. 0.5 cm wax bolus was used for disease involving the anterior commissure (AC). Table 2 summarizes the treatment parameters.

**Table 2:** Treatment parameters

**Parameters**	**Stage T1N0**	**Stage T2N0**
	**(433) Patients (%)**	**(262) Patients (%)**
Field size (cm2)		
< 30.5	215 (49.6)	0
30.5- 35.5	165 (38.1)	106 (40.4)
≥ 35.5	53 (12.2)	156 (59.5)
A. Dose fraction size		
2.5 Gy	177 (40.8)	86 (32.8)
Total dose (Gy)		
55	30 (6.9)	7 (2.7)
57.5	134 (30.9)	63 (24.0)
60	13 (3.0)	16 (6.1)
Tx duration (days)		
30	25 (5.7)	22 (8.4)
31-34	141 (32.5)	45 (17.1)
≥ 35	11 (2.5)	19 (7.2)
BEDcGy_15_ (cGy)		
Median	6520	6580
range	6058- 6820	6160- 6820
B. Dose fraction size		
2.0 Gy	256 (59.1)	176 (67.1)
Total dose (Gy)		
64	52 (12.0)	24 (9.1)
66	202 (46.6)	109 (41.6)
68	2 (0.46)	2 (0.7)
70	0	41 (15.6)
Tx duration (days)		
≤45	48 (11.0)	18 (6.8)
46- 50	203 (46.8)	110 (42.0)
≥ 51	5 (1.15)	48 (18.3)
BEDcGy_15_ (cGy)		
Median	6340	6520
range	6040- 6700	6040- 6910

*Abbreviations*: Tx = treatment; BED = biologically effective dose.

### Field size and set up

#### T1N0

All patients with T1 disease were treated with parallel-opposed fields, to cover the glottic larynx with 1-2 cm margins. The field size was obtained by multiplying the field length by the field height. They ranged from 22- 38.5 cm^2^ (median: 27.5 cm^2^). Typically, the superior border was placed at around the superior border of the thyroid cartilage, the inferior border was placed at around the bottom of the cricoid cartilage, the anterior border extending beyond the skin surface and the posterior border stopping at the anterior border of the vertebral body of cervical spine. Elective nodal irradiation was not given.

#### T2N0

Two hundred and thirty eight (90.8%) of T2 patients were treated with parallel opposed fields. Although the initial field sizes ranged from 35 to 72 cm^2^ (median: 42), 82% of patients were treated with 42 cm^2^ (6 cm x 7 cm) or smaller portals.

Twenty one (8%) patients were treated with a single anterior appositional field, in which electron beam were used after the initial 30 Gy given by 6 MV photon beam. Three (1.1%) patients were treated with a three- field technique by adding an anterior field to two opposing fields.

### Dose and fractionation

Dose was prescribed at the midline along the central axis or recalculated at the ICRU reference point. Between the period of 1983- 1988 and 1996- 2005, patients were treated with a fraction size of 2.0 Gy whereas during 1989- 1995, a fraction size of 2.5 Gy was utilized because of constraints in linear accelerator machine in the hospital.

To allow comparison of dose prescribed in different periods, we opted to compute the tumor biologically effective dose (BED) by using the standard linear quadratic formula (LQ) with time factors corrected [10]:

(1) $$\text{Tumor BED}=nd\left(1+d/\left[\alpha /\beta \right]\right)–{\text{log}}_{\text{e}}\;2\;\left(T–T\text{k}\right)/\alpha T\text{p}$$

where *n* fractions of *d* Gy are given in an overall time of *T* days and kick off time (*T*k) for tumor repopulation. We assume α/β = 15 for laryngeal cancer[11], *T*k = 28 for tumor [12], *T*p = average cell number doubling time during continuing radiation, 3 days for tumor [13]. Alpha (α) = 0.35 Gy^-1^[13][coefficient of non-repairable injury, log cell kill (exponentially-based logs) per gray of dose].

#### T1N0

During 1983-1988 and 1996-2005, the dose-fractionation schedule was 66 Gy in 33 fractions whereas during 1989- 1995, the schedule was 57.5 Gy in 23 fractions.

#### T2N0

During 1983 – 1988, the dose- fractionation schedule was 66 Gy in 33 fractions whereas during 1989-1995, the schedule was 57.5 Gy in 23 fractions. Since 1996, we have prescribed up to 70 Gy in 35 fractions for all T2 disease.

### Follow up and assessment

All patients underwent evaluation of response to treatment by endoscopy examination at 6 to 8 weeks after completion of RT treatment. Patients were regularly seen once every three months during the initial 2 years and then six-monthly up to 5 years and then yearly thereafter.

### Complications

Acute and chronic complications were scored according to the Common Terminology Criteria for Adverse Events version 3.0 [14].

### Statistical analysis

Local and neck failure was defined as clinically/radiological detectable disease in larynx and cervical lymph node (LN) respectively. Distant metastasis (DM) was defined as clinically or radiologically detectable disease outside the larynx and cervical LN. Clinicopathologic parameters that were analyzed included age (<61 vs. 61-70 vs. >71), gender (male vs. female), pre-treatment hemoglobin (Hb) level (<13.0 vs. ≥13.0 g/dl), T sub-stage (T1a vs. T1b) [for T1 disease], impaired cord mobility (yes vs. no) [for T2 disease], tumor grading (well vs. moderate vs. poorly differentiated squamous cell carcinoma) and involvement of AC (yes vs. no). Treatment parameters included dose fraction size (2.0 Gy vs. 2.5 Gy), BEDGy_15_ given (<65.0 Gy_15_ vs. ≥ 65.0 Gy_15_), treatment field size in cm^2^ (< 30.5 vs. 30.5 – 35.5 vs. > 35.5), and treatment period (1983- 1990 vs. 1991- 2000 vs. 2001- 2005).

All time-related events were measured from date of the first RT treatment. The actuarial local control (LC) rate, ultimate LC rate, cause- specific survival (CSS) and overall survival (OS) were calculated by the Kaplan-Meier method. Difference of the endpoints of patient cohorts stratified by various prognostic factors were evaluated by the Log- rank test. Cox proportional hazard model was used for both univariate and multivariate analysis to determine the hazard ratios and significance of potential risk factors for LC and CSS. All statistical tests were two-sided and performed at the 0.05 level of significance (*p* value). Only factors with a level of significance less than 0.05 in univariate analysis would be further analyzed in the multivariate analysis. We used SPSS, version 15.0, (SPSS Inc.,Chicago, IL) for all statistical analyses.

## Results

### Local and neck control

**Figure 1:**
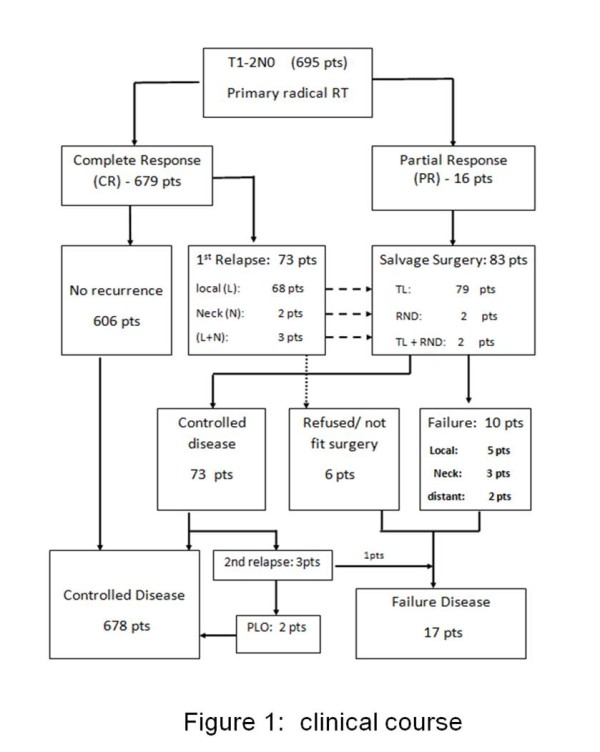
**Clinical course.***Abbreviations:* pts = patients; RT = radiotherapy; TL = total Laryngectomy; RND = radical neck dissection; PLO = pharyngolaryngectomy; 1st = First; 2nd = Second.

**Figure 2:**
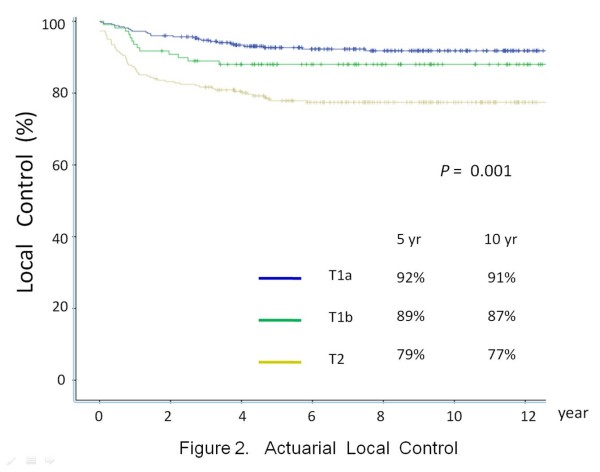
Actuarial local control.

The clinical course of this patient cohort is shown in Figure 1. The median follow- up period was 10.5 years (range: 3.3-26 years). LC after primary RT is depicted in Figure 2. The 5-year and 10-year actuarial LC rates were as follows: T1A, 92% and 91%; T1B, 89% and 87%; T2, 79% and 77% respectively.

Complete response was achieved in 679 (97.6%) patients, while 16 (2.3%) patients (3 T1 and 13 T2) had residual disease at vocal cord(s). Seventy three (10.5%) among the 679 patients who achieved complete response had first relapse with a median time of 15 months. The first sites of relapse included: vocal cord(s) only in 68 (10%) patients (38 T1 and 30 T2), regional LN only in 2 (0.2%) patients (2 T2), vocal cord(s) plus regional LN in 3 (0.4%) patients (3 T2).

Table 3 showed parameters that have prognostic significance on LC. For T1 disease, on multivariate analysis, LC was adversely affected by the poorly differentiated histology (Hazard Ratio [HR]: 7.5, *p* = 0.035), involvement of AC (HR: 2.34, *p* = 0.011), fraction dose size of 2.0 Gy (HR: 2.17, *p* = 0.035) and tumor BED Gy_15_ < 65.0 Gy_15_ (HR: 3.38, *p* = 0.017).

**Table 3:** Univariate and multivariate analysis of factors affecting local control

**T1N0**					**T2N0**			
**Parameters**	**Event/pts**	**Uni-variate**	**Multivariate analysis**		**Event/pts**	**Uni-variate**	**Multivariate analysis**	
		***P*** **value**	**Hazard ratio (95% CI)**	***P*** **value**		***P*** **value**	**Hazard ratio (95% CI)**	***P*** **value**
Age								
<61	18/142	0.302	-	NS	22/90	0.747	-	NS
61-70	15/153				23/103			
>70	9/138				13/69			
Sex								
Male	41/413	0.445	-	NS	55/249	0.831	-	NS
Female	1/20				3/13			
Substage								
T1A	28/324	0.24	-	NS	NA	-	-	-
T1B	14/109							
Grade								
Well diff	9/154	0.0001*	1	0.035*	10/64	0.025*	1	
Moderate diff	29/273		1.91 (1.2- 3.85)		40/179		2.4 (1.7- 3.2)	0.022*
Poorly diff	4/6		7.5 (3.42- 5.24)		8/19		4.3 (2.6- 8.9)	
Impaired Cord mobility								
yes	NA	-	-	-	11/51	0.53	-	NS
No					47/211			
Subglottic Extension								
Yes	NA	-	-	-	23/80	0.038*	2.15 (1.78-5.51)	0.027*
No					35/182		1	
Hb (g/dL)								
< 13.0	6/45	0.367	-	NS	15/28	0.001*	3.78 (1.34-8.40)	0.031*
≥ 13.0	36/388				43/234		1	
AC								
No	14/236	0.004*	1	0.011*	12/76	0.129	-	NS
Yes	28/197		2.34 (1.21-4.52)		46/186			
Field size (cm^2^)								
<30.5	35/215	0.534	-	NS	0	0.138	-	NS
30.5- 35.5	7/165				26/106			
> 35.5	0/53				32/156			
Dose size								
2.0 Gy	32/256	0.021*	2.17 (1.28- 4.18)	0.035*	45/176	0.042*	-	NS
2.5 Gy	10/177		1		13/86			
BEDGy_15_								
< 65.0	36/315	0.025*	3.38 (1.29- 7.83)	0.017*	12/76	0.022*	1.8 (1.2- 5.74)	0.038*
≥ 65.0	6/118		1		46/186		1	
Tx period								
1983- 1990	10/115	0.643	-	NS	17/75	0.336	-	NS
1991- 2000	25/224				29/142			
2001- 2005	7/94				12/45			

*Abbreviations*: pts = patients; Hb = hemoglobin; Tx = treatment; diff = differentiated; AC = anterior commissure; BED = biologically effective dose; * = statistically significant; NS = not significant.

For T2 disease, on multivariate analysis, LC was adversely affected by poorly differentiated grading (HR: 4.3, *p* = 0.022), Hb < 13.0 (HR: 3.78, *p* = 0.031), subglottic extension (HR: 2.15, *p* = 0.027) and tumor BED Gy_15_ < 65.0 Gy_15_ (HR: 1.8, *p* = 0.038).

### Salvage surgery and ultimate control

Table 4 showed the details of the radical salvage surgery performed for the 83 patients with residual disease or first relapses. Ultimate disease control was achieved in 678 (97.5%) patients. The 10-year ultimate LC rates for T1 and T2 disease was 97.6% and 97.4% respectively. Larynx preservation was achieved in 606 (87%) patients.

**Table 4:** Salvage surgery performed

**Stage**	**No of pts**	**1**^**st**^** Failures pts**				**Types of 1**^**st**^** salvage surgery**				**2nd Relapse Pts**	**2nd salvage surgery**	**Ultimate failure Pts (%)**
						**(No. salvaged/No. attempted)**					**(No salvage/attempted)**	
		**local**	**LN**	**Local + LN**	**Total (%)**	**TL**	**RND**	**TL + RND**	**Not fit/refused surgery**			
T1	433	39	0	0	39 (9)	36	0	0	3	7	0/7	10 (2.3)
T2	262	45	2	3	50 (19%)	43	2	2	3	3	2/3 (PLO)	7 (2.6)

Abbreviations: No = number; pts = patients; 1^st^ = first; 2^nd^ = second; LN = lymph node; TL = total Laryngectomy RND = radical neck dissection; PLO = pharynolaryngectomy.

### Survival

**Figure 3:**
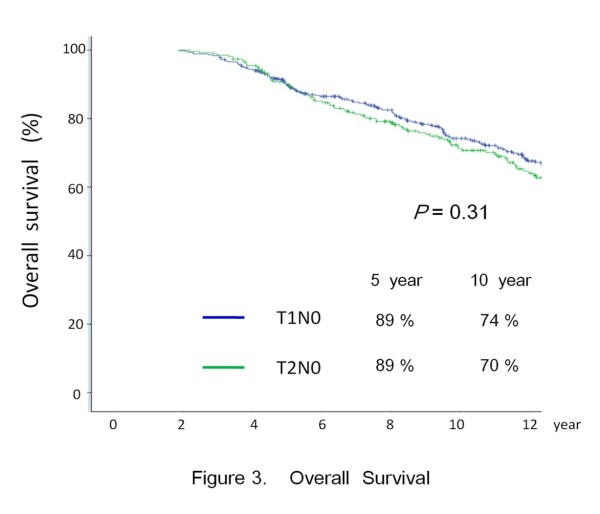
Overall survival.

**Figure 4:**
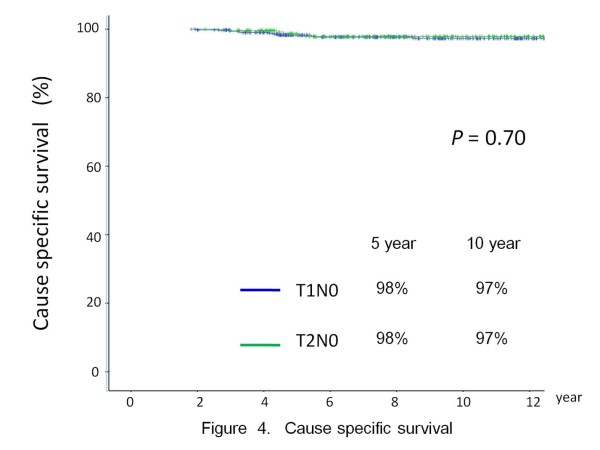
Cause specific survival.

At the last follow up, 272 patients had died: 15 from glottic carcinomas, 118 from second malignancy and 138 from intercurrent disease. Figures 3 and 4 showed the OS and CSS for the entire cohort respectively. For T1 disease, multi-variate analysis showed involvement of AC is the only independent adverse factor for CSS (HR: 3.7, *p* = 0.045). For T2 disease, haemoglobin <13.0 is the only independent adverse factor for CSS (HR: 2.8, *p* = 0.028) [Table 5].

**Table 5:** Univariate and multivariate analysis of factors affecting cause specific survival

**T1N0**					**T2N0**			
**Parameters**	**Event/pts**	**Uni-variate**	**Multivariate analysis**		**Event/pts**	**Uni-variate**	**Multivariate analysis**	
		***P*** **value**	**Hazard ratio (95% CI)**	***P*** **value**		***P*** **value**	**Hazard ratio (95%CI)**	***P*** **value**
Age								
<61	4/142	0.897	-	NS	0/90	0.224	-	NS
61-70	3/153				3/103			
>70	3/138				2/69			
Sex								
Male	10/413	0.459	-	NS	5/249	0.61	-	NS
Female	0/20				0/13			
Substage								
T1A	7/324	0.53	-	NS	NA	-	-	-
T1B	3/109							
Grade								
Well diff	3/154	0.825	-	NS	1/63	0.721		NS
Mod diff	7/273				3/179			
Poorly diff	0/6				1/20			
Impaired Cord mobility								
yes	NA	-	-	-	1/51	0.84		NS
No					4/211			
Subglottic Extension								
Yes	NA	-	-	-	2/80	0.56	-	NS
No					3/182			
Hb (g/ dL)								
< 13.0	1/45	0.76	-	NS	1/28	0.022*	2.8 (1.5-7.8)	0.028*
≥ 13.0	9/388				4/234		1	
AC								
No	2/236	0.026*	1	0.045*	1/76	0.712	-	NS
Yes	8/197		3.7 (1.20-18.5)		4/186			
Field size (cm^2^)								
<30.5	6/217	0.642	-	NS	0	0.42	-	NS
30.5- 35.5	4/165				2/106			
> 35.5	0/53				3/156			
Dose size								
2.0 Gy	7/256	0.463	-	NS	4/176	0.513	-	NS
2.5 Gy	3/177				1/86			
BED Gy_15_								
< 65.0	8/315	0.661	-	NS	4/175	0.534	-	NS
≥ 65.0	2/118				1/87			
Tx period								
1983- 1990	1/115	0.287	-	NS	0/75	0. 334	-	NS
1991- 2000	6/224				4/142			
2001- 2005	3/94				1/45			

*Abbreviations*: pts = patients; Hb = hemoglobin; Tx = treatment; diff = differentiated AC = anterior commissure; BED = biologically effective dose; * = statistically significant; NS = not significant.

### Complications

Regarding acute radiation toxicity, there was no incidence of grade 3 or higher acute complications in T1 disease. For T2 disease, 9 (1.2%) patients experienced grade 3 acute radiation toxicity, with an incidence of 9/86 [10.4%] in patients treated with 2.5 Gy compared with none in those treated with 2.0 Gy (chi-square test value: *p* = 0.001). Eight of them had severe odynophagia and grade III confluent mucositis, requiring nasogastric tube feeding or admission for intravenous fluid supplement. All of them needed treatment interruption of more than 4 treatment days. One patient developed grade IV laryngeal edema and necessitated temporary tracheostomy during the last week of RT and ultimately treatment was prematurely terminated after 55 Gy delivered over 22 fractions. There is no treatment death related to acute radiation toxicity. For chronic complication, no clinical or radiological chondroradionecrosis that warrants laryngectomy has been observed.

## Discussion

The debate on the optimal treatment options for early GC has a long history, mainly because of absence of definitive prospective randomized trials for comparing the various treatment options [15]. A survey conducted in eleven regions/countries in Asia revealed that in regions following the ‘British school’ like Hong Kong and Singapore, RT alone has remained the primary treatment modality for early laryngeal cancers [4]. Vlantis et al. reviewed the treatment results of laryngeal carcinoma in a single institution in Hong Kong [16]. For early stage (T1 & T2) glottic lesions, primary RT remained their mainstay of treatment.

As laser surgery become more popular since Steiner’s landmark report [17], it is expected that it will be more popular in local localities.

Focusing on primary irradiation, in many RT centers, Cobalt 60 or 2-4 MV X-rays machines have been decommissioned. It is anticipated that 6 MV photon beams generated by LA will become the prevailing workhorse for treatment in clinical practice [18]. Table 6 summarizes the published outcome results of early GC treated with 6 MV photons.

**Table 6:** Published local control and survival rate of early GC treated with 6 MV definite radiotherapy

**Author (year)**	**Patient number**	**Local Control (%)**	**OS (%)**	**CSS (%)**
[ref]		5 year*, 10 year #	5 year *, 10 year#	5 year*, 10 year#
Warde et al. [5]	T1 + T2: 72	T1a: 91*	T1 + T2: 75.8*	NA
		T1b: 82*		
		T2: 69*		
Lee et al. [18]	T1: 86	T1a: 82.2*	T1: 85*	NA
	T2: 29	T1b: 76.2*	T2: 74*	
		T2: 68.1*		
Franchin et al. [23]	T1: 323	T1: 90#	T1: 66#	NA
	T2: 87	T2: 78#	T2: 55#	
Cellai & Frata et al. [6,7]	T1: 35	T1: 84*, 83#	T1: 77*, 57#	T1: 95*, 93#
	T2: 68	T2: 73*, 70#	T2: 59*, 37#	T2: 89*, 86#
Chera et al. [8]	T1 + T2: 23	T1a: 94*, 93#	T1 a: 82*, 62#	T1a: 97*, 97#
		T1b: 93*, 91#	T1b: 83*, 57#	T1b: 99*, 95#
		T2a: 80*, 80#	T2a: 76*, 51#	T2a: 94*, 93#
		T2b: 70*, 67#	T2b: 78*, 49#	T2b: 90*, 84#
Current series	T1: 433	T1a: 92*, 91#	T1: 89*, 74#	T1: 98*, 97#
		T1b: 89*, 87#	T2: 89*, 70#	T2: 98*, 97#
	T2: 262	T2: 79*, 77#		

*Abbreviations*: yr = year; NA = not available; OS = overall survival; CSS = cause specific survival.

There have been concerns about possible tumor under-dosage when using 6MV or higher energy photons for early GC. It has been reported that the lack of electronic equilibrium at the air- tissue interface in the laryngeal air cavity is more pronounced with both higher- energy X rays and with small field size [19]. The other issue is the lack of adequate buildup tissue in the area where the neck is thin [20]. Izuno et al. observed a significant inferior 5-year LC rate for T1N0 GC patients when treated with 8/10-MV photons compared to Co60 (60% vs. 88%)[21].

### Factors affecting local control

#### T1 disease

The factors of AC involvement, fraction dose size and time dose factor affecting the LC rate of T1 disease has been discussed in a separate article [22].

#### T2 disease

As noted, the LC rate of T2 disease treated with fractional dose size of 2.5 Gy was superior to those treated with fraction size of 2.0 Gy. However, this significance is lost in multi-variate analysis. We believe that the tumor BEDGy_15_ is the key factor to explain the significant difference which is confirmed by the multi-variate analysis.

As shown in Table 7, the fractionation schedule of 2.5 Gy x 23 daily fractions can achieve a tumor BEDGy_15_ ≥ 65 Gy_15,_ however, it can also be associated with a significant higher incidence of grade 3 acute radiation toxicity as shown by the higher BEDGy_10_ for acute mucosa and larger treatment field size in T2 disease.

**Table 7:** Tumor, acute mucosal and late normal BED for two fractionation scheme

**Dose size (Gy)**	**Fraction number**	**Total dose (Gy)**	**Overall treatment time (OTT) in days**	**Tumor BED (Gy**_**15**_**)**	**Acute Mucosal BED(Gy**_**10**_**)(aim 59-63 Gy**_**10**_**)**[13]	**Late normal BED (Gy**_**3**_**)(aim <117 Gy**_**3**_**)**[13]
2.0	35	70	47	66.79	52.32	117
2.5	23	57.5	31	65.28	52.87	105.4

*Abbreviations:* BED *=* biologically effective dose.

Unlike our recommendation of using a fraction size of 2.5 Gy for T1 disease [22], our current practice for T2 disease is to prescribe 70 Gy in 35 fractions, 2 Gy per fraction, 5 daily fractions per week. This can achieve a tumor BED ≥ 65 Gy_15_ without an excess of grade 3 acute mucosal toxicity.

Anaemia, poor differentiation of tumor grade and subglottic extension are known reported adverse prognostic factors in LC of GC [5,7,8] .

The impact of impaired cord mobility on LC of T2N0 GC is still controversial. Our data is consistent with other published results that reported no prognostic significance on LC [23,24] . However, other studies, including a meta-analysis did suggest its adverse impact on LC [7,25,26].

In view of the relatively inferior LC rate of T2 compared with T1 disease, more intensive treatment strategies are being explored. The role of bid hyperfractionation schedule will become clear when the mature result of RTOG 95 -12 study become available. Another approach is the use of concurrent chemotherapy. Nevertheless, there are some data showing improvement of LC by platinum/5FU based regimens [27,28] but others showed conflicting results [29,30].

There are still controversies over the use of Intensity- Modulated Radiotherapy (IMRT) for early stage GC. Feigenberg et al. warned about the treatment failure seen in patient with early GC who received definite RT with IMRT technique [31]. Their concerns were mainly errors due to contouring errors and organ motion.

Some technical studies revealed IMRT technique permit more irradiation dose sparing at carotid arteries and might have favorable implication on long term risk of cerebrovascular accident [32,33]. However, others still continue to recommend conventional RT fields and technique for treatment of patient with early stage GC [34,35].

## Conclusion

To the best of our knowledge, our report is the largest study on RT outcomes in early GC primarily treated with 6 MV photons. Both the LC and CSS rates of T1 and T2 disease in our series are comparable with other reported data. This reassures the efficacy of 6 MV photons used in primary RT for early GC. Nevertheless, the results of the current report is a retrospective, single institution study and therefore can be subjected to biases, especially other known significant prognostic factors including tumor volume and smoking details are lacking. Prospective measurement of voice quality is also not available in this retrospective review.

## Competing interests

The authors declare that they have no competing interests.

## Authors’ contributions

CCT participated in the study’s design and coordination, performed acquisition of data and drafted the manuscript. KHA and FYC participated in data analysis and revised the manuscript. RKCN and SMC participated in study’s design and revised the manuscript. JSKA, YTF and SCKL revised manuscript critically for important intellectual content. All authors read and approved the final manuscript.
